# Combining functional genomics strategies identifies modular heterogeneity of breast cancer intrinsic subtypes

**DOI:** 10.1186/1756-0381-7-27

**Published:** 2014-12-29

**Authors:** Nima Pouladi, Richard Cowper-Sallari, Jason H Moore

**Affiliations:** Departments of Genetics and Community and Family Medicine, Institute for Quantitative Biomedical Sciences, One Medical Center Dr, Lebanon, NH 03756 USA; The Geisel School of Medicine, Dartmouth College, One Medical Center Dr, Lebanon, NH 03756 USA

**Keywords:** Breast cancer subtype, Heterogeneity, *β*-diversity, Gene module

## Abstract

**Background:**

The discovery of breast cancer subtypes and subsequent development of treatments aimed at them has allowed for a great reduction in the mortality of breast cancer. But despite this progress, tumors with similar characteristics that belong to the same subtype continue to respond differently to the same treatment. Five subtypes of breast cancer, namely intrinsic subtypes, have been characterized to date based on their gene expression profiles. Among other characteristics, subtypes vary in their degree of intra-subtype heterogeneity. It is not clear, however, whether this heterogeneity is shared across all tumor traits. It is also unclear whether individual traits can be highly heterogeneous among a majority of homogeneous traits.

**Results:**

We employ network theory to uncover gene modules and accordingly consider them as tumor traits, which capture shared biological processes among the subtypes. We then use the *β*-diversity metric from ecology to quantify the heterogeneity in these gene modules. In doing so, we show that breast cancer heterogeneity is contained in gene modules and that this modular heterogeneity increases monotonically across the subtypes. We identify a core of two modules that are shared among all subtypes which contain nucleosome assembly and mammary morphogenesis genes, and a number of modules that are specific to subtypes. This modular heterogeneity, which increases with global heterogeneity, relates to tumor aggressiveness. Indeed, we observe that Luminal A, the most treatable of subtypes, has the lowest modular heterogeneity whereas the Basal-like subtype, which is among the hardest to treat, has the highest. Furthermore, our analysis shows that a higher degree of global heterogeneity does not imply higher heterogeneity for all modules, as Luminal B shows the highest heterogeneity for core modules.

**Conclusions:**

Overall, modular heterogeneity provides a framework with which to dissect cancer heterogeneity and better understand its underpinnings, thereby ultimately advancing our knowledge towards a more effective personalized cancer therapy.

**Electronic supplementary material:**

The online version of this article (doi:10.1186/1756-0381-7-27) contains supplementary material, which is available to authorized users.

## Background

Breast cancer is the most common cancer in women worldwide [1]. The discovery of breast cancer subtypes and subsequent development of treatments aimed at each of the subtypes has allowed for a great reduction in the mortality of breast cancer [2–4]. But despite this progress, tumors with similar characteristics continue to respond differently to the same treatment [5]. It is therefore imperative to continue dissecting the heterogeneity of breast cancer [4].

**Figure 1:**
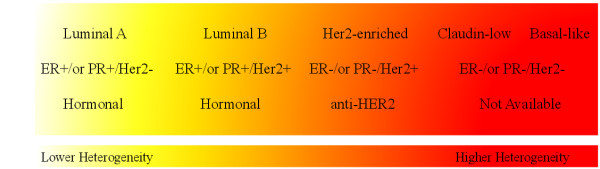
**The relative degree of heterogeneity of various breast cancer subtypes.** The intrinsic subtypes have been ranked from the left (yellow) to the right (red) according to their heterogeneity degrees in which Luminal A and Basal-like are poorly and highly heterogeneous, respectively. The second row shows the overlap of the intrinsic based subtype classification with that of IHC based. The last row shows the availability of targeted therapy for each subtype. The relatively more heterogeneous Basal-like and Claudin-low are the subtypes with very poor prognosis since no therapeutic has been tailored to their biology thus far (See the text).

Breast tumor heterogeneity can be defined as variation among patients [6]. Five subtypes of breast cancer have been characterized to date based on their gene expression profiles [7]. Named the intrinsic subtypes they are: Luminal A, Luminal B, HER2-enriched (also called HER2-related), Claudin-low and Basal-like. Breast tumors can also be classified based on the immunohistochemical profile (IHC) of three key receptors: the estrogen receptor (ER), progesterone receptor (PR), and human epidermal growth factor receptor 2 (HER2). The four IHC based subtypes are: ER-/PR-/HER2- (triple-negative), ER-/PR-/HER2+, ER+/ or PR+/HER2+, and ER+/ or PR+/HER2-. IHC-based and intrinsic subtypes overlap (Figure 1). The first IHC-based subtype overlaps with Basal-like and Claudin-low intrinsic subtypes, the remaining three overlap HER2-enriched, Luminal B and Luminal A, respectively [6]. Subtypes range in aggressiveness. Basal-like, Claudin-low, HER2-enriched and Luminal B tumors are significantly more aggressive than Luminal A tumors [7], with Basal-like and Claudin-low at the top of the ranks. Basal-like and Claudin-low are part of triple-negative breast cancers, which currently for them there is no effective therapy (Figure 1) [4, 8–10]. The subtype of a tumor will therefore inform the clinicians of the course of action and determine the patient’s odds of survival [4, 11, 12].

Breast cancer subtypes also vary in their degree of intra-subtype heterogeneity; more aggressive subtypes are more heterogeneous. Harrell et al. assessed the degree of heterogeneity of 298 different breast cancer gene expression signatures across the five intrinsic subtypes (Figure 1) [13]. By using pooled gene signatures they were able to show that all subtypes are more heterogeneous than normal breast tissue and that the Basal-like subtype is the most heterogeneous. Based on the immunohistochemical classification, ER-negative breast tumors, which are composed primarily of the Basal-like tumors, were also found to be significantly more heterogeneous than the rest of histological subtypes [8, 14].

However, what remains unclear from these observations is whether particular subtypes, such as ER-negative or Basal-like tumors, are more heterogeneous across all of their tumor traits such as proliferation ability or angiogenic potential. Conversely, it is also unclear whether subtypes with less heterogeneity at the whole transcriptome level, such as Luminal A, could show increased heterogeneity in specific traits. Finally, there could also be a subset of the transcriptome that is heterogeneous for all breast tumors and would thus constitute a core source of cancer variability. The focus of this study is to explore the distinction between global and local transcriptome heterogeneity. To do so we develop a framework that makes use of network theory to build and characterize gene modules and of ecological measures of diversity with which to quantify and compare global and local heterogeneities across the five intrinsic subtype of breast cancer.

Namely, we employed *β*-diversity as the measure of heterogeneity [15]. Park et al. also adapted the Shannon and Simpson diversity indices from ecology to measure the degree of intra-tumor genetic heterogeneity of invasive breast carcinoma in 8q24 copy number data [16]. We used *β*-diversity for its intuitive derivation and interpretability but most importantly for its ability to make simultaneous comparison of multiple groups by inheriting the power of ANOVA [15]. We refer to this framework as ‘modular heterogeneity’.

## Methods

### Data

We used the breast microarray gene expression data that has been published by Harrell et al. [13], and can be retrieved from the repository of UNC Microarray Database by searching for the name of its authors: https://genome.unc.edu/cgi-bin/SMD/publication/viewPublication.pl?pub_no=107. This data set has some advantages over other breast cancer data sets. It was shown that variation of expression values of genes in this data set stems from the biology and not from cohort/ source or 7 Agilent microarray platforms [13]. It contains a compendium of normal breast epithelium and different subtypes of breast cancer. Also, all of the samples had been processed in the same lab. We pre-processed the data according to Harrell et al. and we averaged the normalized log 2 ratio of the probes mapped onto the same gene [13]. The probes without mapping onto any gene symbol were discarded. This process resulted in 13,822 genes. We focused our downstream analysis on 286 unique samples out of 414 ones. They include Normal breast tissues, Claudin-low, HER2-enriched, Basal-like, Luminal A and Luminal B, Metastatic Claudin-low, Metastatic HER2-enriched, Metastatic Basal-like, Metastatic Luminal A, and Metastatic Luminal B breast tumor subtypes, which for them 17, 42, 22, 31, 80, 45, 8, 13, 17, 6, 5 samples available, respectively. Afterwards, we quantile normalized the 286 selected arrays by employing library limma implemented in R in order to make experiments comparable with each other. We chose quantile normalization for between array normalization for its high efficacy. Also, studies with focus on investigating the variance of gene expression in microarray experiments compared the effect of different between array normalization techniques, and finally employed the quantile normalization in their downstream analysis [17]. Then, median absolute deviation (MAD) of expression values of all the genes across all the samples were calculated and 2,511 transcripts with MAD greater than the Upper Quartile Q3 were selected and used in the rest of the analysis.

### ***β***-diversity

We utilized the concept of *β*-diversity as a measure of heterogeneity of each phenotypic state of breast. It is defined as the variability in species’ composition among sampling units for a given area at a given spatial scale [15]. Also, the relative abundance of species can be incorporated into it. It is calculated by taking the average distance (or dissimilarity) from an individual unit to the group centroid, using an appropriate dissimilarity measure [15, 18]. *β*-diversity is quite flexible as any meaningful distance measure can be adapted to it. Most importantly, simultaneous comparison of heterogeneity among several different areas or groups is possible. Briefly, a null statistical model stating that there is no difference among heterogeneity of sampling units across different regions is defined. Afterwards, ANOVA test on the computed distance of each individual to its corresponding group spatial median or centroid in the full dimensional space of species is employed in order to reject the null hypothesis at the significance level of interest, with either permutation or traditional *F* ratio test. This distance based ANOVA is called multivariate analysis of dispersion [19], which is also capable of addressing a few common problems in biological experiments such as failure of normality requirement of variables, and higher number of variables than that of samples [19]. Method ‘betadisper’ implemented in R library vegan together with its associated methods has implemented multivariate analysis of dispersion.

#### Global transcriptome heterogeneity

We computed the *β*-diversity values of all of the phenotypic states by including all the transcripts, as measures of their global transcriptome heterogeneity. In order to do so, the expression values of 2,511 transcripts were first robust standardized. Consequently, the median absolute deviation and median of each transcript became one and zero, respectively, across all samples compiled together [20]. This guaranties that expression values of each gene contributes equally to the variation in the calculated pair-wise Euclidean distances among samples. We chose Euclidean distance for it has intuitive meaning and is the most common distance metric in genomics. Afterwards, the new distance transformed variables were subjected to multivariate analysis of dispersion. In the next step, we performed 30 pair-wise comparisons in order to examine the significance of difference in heterogeneity among different phenotypic states; 5 for normal versus cancerous states, 5 for cancerous versus their corresponding metastatic states, 10 for cross cancerous states, and 10 for cross metastatic states. We performed 10,000 permutations on the residuals in order to have P-values at 0.01 level of significance [21]. Finally, we FDR adjusted all of the 30 calculated P-values to correct for multiple comparisons.

#### Local transcriptome heterogeneity

We calculated the *β*-diversity values for each module to quantify the degree of the heterogeneity at the local level (each module) for various phenotypic states. Therefore, the expression values of the transcripts composing each module were first separately robust standardized [20]. Then, pair-wise Euclidian distances across all of the samples were computed for each module, separately. Afterwards, the new distance transformed variables were subjected to multivariate analysis of dispersion. We performed 30 pair-wise comparisons per each module, the same way as we did for assessment of global transcriptome heterogeneity. Finally, all of the 240 calculated P-values (30 × 8 modules) based on 10,000 permutations on the residuals were FDR adjusted to correct for multiple comparisons.

### Weighted gene co-expression network

We employed methodology developed by Zhang et al. [22] in order to construct the global breast weighted co-expression network. Further details about the described methodology have been elaborated in [23]. In the following two sections, we provide the reader with an overall process and how it has been utilized in this study. Library WGCNA implemented in R (Weighted Gene Co-Expression Network Analysis) was used in order to construct the breast weighted gene co-expression network [24].

#### Constructing the breast cancer global network

A gene co-expression network is defined as a group of genes, which represent the nodes of the network. The edges between each pair of nodes are formed given their corresponding genes are co-expressed. Correlation coefficient between expression values of each pair of genes can serve as a proxy for measuring the strength of co-expression between each pair of genes. Among the various correlation coefficients, bi-weight mid-correlation has some advantages which makes it a suitable choice for co-expression analysis of microarray gene expression data. It inherits the high power of Pearson correlation on one hand, and the high robustness of Spearman correlation on the other hand [23]. Therefore, signed bi-weight mid-correlation coefficients were calculated for all pairs of genes across all the samples lumped together and the corresponding correlation matrix was formed consequently. Afterwards, this correlation matrix was turned into a similarity matrix, *S*=[*s*_*ij*_] with this transformation,  in which *cor*(*i*,*j*) stands for the correlation coefficient between pair of genes *i* and *j*. This transformation makes the entries of *S* fall in domain [0,1].

Next, the constructed similarity matrix was transformed into a weighted adjacency matrix, *A*=[*a*_*ij*_] which each of its entries measures the strength of each between node connection. This can be done by employing power adjacency function *a*_*ij*_=*abs*(*s*_*ij*_)^*β*^ in which the power *β* is called a soft threshold. This technique is known as soft-thresholding because the edges of final network will be weighted instead of being binary. On the other hand, soft-thresholding saves the continuity of measured correlation coefficients. The right choice of parameter *β* is important. The power *β* is chosen in such away that the frequency distribution of the connectivity of nodes approximates scale free topology, which is a biologically plausible assumption [25]. Recall that connectivity of each node is defined as the sum of its weighted connections to other nodes, . Then the square of the correlation between logarithm of connectivity distribution, log(*p*(*k*)), and that of connectivity, log(*k*), is defined as scale-free fitting index (*R*^2^). This index tells us how well the frequency distribution of connectivity of nodes approximates scale free topology. Networks with *R*^2^ closer to 1 estimates scale free topology criterion to a better extent. Thus, the computed similarity matrix was raised to different values of *β* spanning a range from 1 to 30 and their corresponding *R*^2^s were calculated. By drawing computed values of *R*^2^ against their corresponding *β*s, we noticed that scale-free fitting index of *R*^2^ curve reached its saturation point at power 13 with *R*^2^=0.9. The power of 13 is also the number that developers of the method had suggested. Therefore, we raised the computed similarity matrix to power 13, and constructed the adjacency matrix of breast weighted gene co-expression network.

#### Module identification

In order to find subset of genes (module) that are tightly connected with each other, the distances between all pairs of genes are calculated based on the adjacency matrix *A*. Afterwards, the computed distance matrix is subjected to a clustering method which results in detecting the modules. As a measure of node similarity, which is used subsequently to make a distance matrix, topological overlap [26] between genes is a reasonable choice. Topological overlap measure between a pair of genes assesses the relation of each pair of genes with the rest of the genes across the network in contrast with adjacency matrix in which this feature is missing. It is simply the normalized version of the number of shared neighbors between a pair of nodes in a graph. Plus, it is a robust measure that filters the effect of noisy edges with low signal, and has been successfully utilized in biology [22, 23]. Thus, we transformed the computed adjacency matrix *A* into a topological overlap matrix (*TOM*) and subsequently into a distance matrix, 1−*TOM*. Then, average linkage hierarchical clustering was applied to the calculated distance matrix. Finally, ‘Dynamic Hybrid’ cutting algorithm [27], which has been successfully employed in other studies [28, 29], was utilized in order to cut branches off the dendrogram, thus giving rise to detecting the modules. Consequently, we found 8 different gene co-expression modules, and used them in our downstream analysis. Note that according to the described methodology, a gene co-expression module is defined as a subset of genes with high topological overlap. Different modules were labeled with different colors in order to be distinguished from each other.

### Gene ontology analysis

We employed Gorilla [30], http://cbl-gorilla.cs.technion.ac.il/, in order to infer what biological process each module contributes to. All of the 2,511 genes used in this study were considered as reference background gene list. Each module was then separately analyzed against the reference gene list.

## Results

### Global heterogeneity

**Figure 2:**
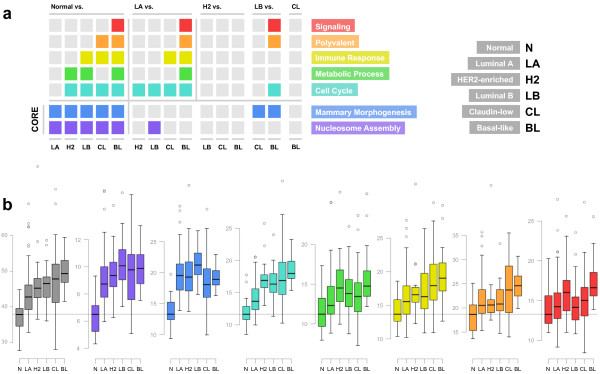
**Alteration of global and modular** ***β*** **-diversity values in distinctive phenotypic states of breast tissue.** **a** Colored matrix representing 105 out of the 240 pair-wise comparisons performed in this study. The colored cells represent tests with FDR corrected P-values < 0.01. Subtype comparisons are ordered based on global *β*-diversity. Modules are ordered based on the number of subtypes in which they exhibit significantly higher *β*-diversity than normal breast tissue. Notably purple and blue modules significantly show larger *β*-diversity in all of the phenotypic states of breast tumor compared to that of normal state. The pink module has been removed from this matrix. The corresponding metastatic states are not shown since none of the subtypes at this state shows significantly different levels of *β*-diversity when compared to their cancerous counterparts or among themselves (See the text). **b** Box plots corresponding to the patterns of *β*-diversity across subtypes. Gray box plots correspond to global *β*-diversity for the available transcriptome. Colored box plots correspond to modules as indicated in the legend in panel a. Each box plot depicts the distribution of Euclidean distances between patients and their corresponding subtype spatial median (See the text).

Before delving into the modular analysis of breast cancer heterogeneity, we first measured the *β*-diversity across the available transcriptome (2,511 transcripts) to assess the global transcriptome heterogeneity for all subtypes. We found an increment in *β*-diversity from normal to Basal-like states (Figure 2b; gray). Basal-like having a significantly higher *β*-diversity than the Luminal subtypes (corrected P-value < 0.01) but only slightly higher than those of Claudin-low and HER2-enriched. Transition from cancer to metastatic stage showed only a minimal increase in global transcriptome *β*-diversity and once at the metastatic level, all subtypes showed a similar values (Additional file 1: Table S1). Our assessment of global transcriptome heterogeneity using *β*-diversity is largely consistent with the findings of Harrell et al. [13].

### Network construction and module composition

In order to assess the modular nature of transcriptome heterogeneity we partitioned the available transcriptome into co-expressed gene modules. We used data from all stages (normal, cancer and metastatic) and subtypes (286 samples) independently of tumor heterogeneity so as to make our modules comparable between subtypes. We used co-expression modules as a proxy for tumor traits for two reasons. First, correlation among gene expression patterns has been used to effectively capture underlying regulatory and signaling circuits [31]. Second, co-expression modules are conserved across species and are organized into coherent functions [32, 33]. We determined our co-expression modules by building a global breast cancer transcriptional network using weighted gene co-expression analysis [22]. This method has been widely used and has a good ability to recapitulate empirical results [28, 34–37]. Additionally, it is computationally tractable but does not trivially reduce the problem of network inference to pair-wise correlations between genes. Applying a Dynamic Hybrid cutting algorithm to the network yielded eight modules based on high topological overlap (See Methods for additional details).

We characterized the modules with gene ontology (GO) analysis. The genes constituting each module were compared to a background gene list consisting of all 2,511 genes in the study (Additional file 2: Table S2). Four modules were significantly enriched for GO biological process terms (P-value < 0.01; FDR corrected). Three of these had fully coherent terms. We refer to them by their GO terms as the nucleosome assembly (colored purple, 109 unique genes), immune response (yellow, 369) and cell cycle (turquoise, 329) modules. The fourth of these modules was enriched for extracellular matrix organization, developmental processes, biological adhesion, and angiogenesis among others; we refer to this module as the polyvalent module (orange, 620). Another three modules were enriched but did not satisfy multiple testing. Their enriched GO terms, however, did reflect a coherent function. We refer to these modules as mammary morphogenesis (blue, 509), signaling (red, 241) and metabolic process (green, 228) modules. One module remained uncharacterized (pink, 67). Table S3 in Additional file 3 contains the names of all of the genes constituting these modules.

### Modular heterogeneity

Having defined a set of modules with which to distinguish between global and local differences we were then able to quantify the degree of *β*-diversity for each module in each subtype and perform all pair-wise comparisons between the five intrinsic subtypes and normal breast tissue (Figure 2a and Additional file 4: Table S4) We performed a total of 30 pair-wise comparisons per module across all stages and subtypes. When compared to normal tissue, we detected a change in *β*-diversity in at least one subtype for all modules except the uncharacterized module (pink). Because this module showed no biological enrichment and no change in any pair-wise comparison we obviated it from further results and discussion, it will however continue to contribute to multiple test correction. As expected, we observed that the number of modules with higher *β*-diversity than normal breast tissue increases monotonically for subtypes with increasingly large global *β*-diversity (Figure 2a; top left). In this sequence of increasing global *β*-diversity, the first two modules to change are nucleosome assembly and mammary morphogenesis in Luminal A. The cell cycle and metabolic process modules follow for HER2-enriched, then the immune response module is added in Luminal B, followed by the polyvalent module in Claudin-low and finally the signaling module, which is exclusive to Basal-like. The notable exception to this trend is the metabolic process module, which does not have higher *β*-diversity in Claudin-low. Compared to normal breast epithelium as the healthy reference phenotypic state, the only modules with increased heterogeneity in all of the breast cancer subtypes are nucleosome assembly and mammary morphogenesis. We thus call them the core breast cancer modules (Figure 2a; bottom left).

However, when we compared the subtypes to each other, this pattern of gradual accretion of high *β*-diversity modules transitioned into an array of modular combinations of high *β*-diversity modules. Notably, Luminal B is the only subtype that varies significantly within the core modules (Figure 2a; bottom right). It has the highest *β*-diversity in the nucleosome assembly module, which is higher than that of Luminal A, and in the mammary morphogenesis module, which is higher than those for Claudin-low and Basal-like (Figure 2b; purple and blue).

Using Luminal A as a reference, the least aggressive subtype with the lowest *β*-diversity, we see that only the first non-core module, cell cycle, shows an increase in *β*-diversity for all non-Luminal A subtypes (Figure 2a; top middle, turquoise). All non-core modules show higher *β*-diversity for Basal-like and Claudin-low differs significantly for the immune response module (Figure 2a; top middle). The pattern observed for the Lumina A comparisons resembles a sparser version of the pattern we see in the Normal tissue comparisons. Finally, in the rest of pair-wise comparisons, only the Basal-like subtype is able to distinguish itself from Luminal B in the cell cycle, polyvalent and signaling modules (Figure 2a; top right). Metastases derived from all five intrinsic subtypes did not show significantly different levels of *β*-diversity when compared to their cancerous counterparts or among themselves (Additional file 5: Figure S1).

With regards to the trends for each module, core modules show a sharp difference between all subtypes and normal tissue but then plateau across the subtypes with the exception of the Luminal B module, which is above the rest (Figure 2b; purple and blue). The cell cycle module, which is the first non-core module, exhibits a similar plateau with the exception of the Luminal A subtype which is closer to normal (Figure 2b; turquoise). It is also the first module in which Basal-like exhibits its characteristic higher *β*-diversity. The metabolic process module shows an intermediate behavior between core and non-core modules, although in this instance it is the HER2-enriched module which breaks away from the group and is comparable in *β*-diversity to Basal-like (Figure 2b; green). The next module in the modular progression, immune response, shows a gradual increase from normal tissue to Basal-like (Figure 2b; yellow). Finally, the last two non-core modules show opposite patterns to the core modules. Where the majority of subtypes have *β*-diversities similar to normal-tissue, Claudin-low and Basal-like in the polyvalent module and Basal-like, and HER2-enriched to some extent, in the signaling module show the highest *β*-diversity.

## Discussion

We have shown that breast cancer heterogeneity is contained in gene modules and that this modular heterogeneity increases monotonically across the five intrinsic subtypes of breast cancer. We found a core of two modules that are shared among all subtypes and a number of modules that are particularly heterogeneous in specific subtypes. This modular heterogeneity increases with global heterogeneity, which in turn relates to tumor aggressiveness. Indeed, we observe that Luminal A, the most treatable of subtypes, has the lowest modular heterogeneity (two out of seven) whereas the Basal-like subtype, which is among the hardest to treat, has the highest (seven out of seven). Furthermore, our analysis shows that a higher degree of global heterogeneity does not imply higher heterogeneity for all modules. Basal-like and Claudin-low subtypes have the highest global heterogeneity yet, for the core module mammary morphogenesis, Luminal B is significantly more heterogeneous than both. We were unable, however, to detect significant changes in modular heterogeneity for the metastatic tumors and only observed minimal increments. This may be due to an absence of power due to the small number of metastatic samples in our study.

The functional enrichments of these modules recapitulate some of the biological processes which play important roles in the biology of breast cancer. The first of the core modules, nucleosome assembly, contains many histone genes. Alteration of chromatin assembly has been shown to play an important role in the progression of breast cancer and is concordant with the observation that global epigenomic changes underlie the heterogeneity of tumors [38–41]. The second of the core modules, mammary morphogenesis, contains FOXA1 (forkhead box protein A1), ESR1 (estrogen receptor 1), AR (androgen receptor) and WNT4 (wingless-type mmtv integration site family, member 4). All of these genes play important roles in the healthy physiology of breast tissue and, when deregulated, in the pathogenesis of breast cancer [39, 42–46].

The first of the non-core modules, cell cycle, shows higher heterogeneity in all subtypes except in Luminal A. This module highlights the clinical difficulty of targeting the cell cycle with cytotoxic agents due to most tumor’s high heterogeneity. Triple-negative tumors, which are primarily of the Basal-like intrinsic subtype, have the highest heterogeneity for this module. A subgroup of these patients does not develop pathologic complete response despite the fact that this subtype has initial responsiveness to chemotherapy. This might be partly as a result of observed high heterogeneity of cell cycle [10, 47]. Another non-core module, immune response, is known to play a particularly critical role in the progression of breast tumors. A correlation has been observed between the activity of immunity-related genes and patient survival for the more aggressive intrinsic subtypes, or ER-negative subtypes, which show a high heterogeneity for this module [48, 49]. Because of its gradual increase in heterogeneity across all subtypes this module is particularly well suited for further refinement of tumor classification. Indeed, immunity-related genes have already been used to classify patients [50].

Finally, the polyvalent and signaling modules sit at the aggressive end of the modular spectrum and are only heterogeneous in the subtypes that are most intractable. Functional enrichments for these two modules reveal biological processes such as extracellular matrix organization, cell adhesion, angiogenesis, cell migration, cell junction organization, synaptic transmission, fluid transport and G-protein coupled receptor signaling; all of which, despite their clear disparity, point to a common theme of cellular interactions and tumor-stroma reaction, whereas the first four modules in the modular progression point toward more inherent cell properties. This particular class of cellular interaction heterogeneity might be responsible for the recently reported inefficacy of angiogenic inhibitors in these subtypes [51]. Furthermore, the signaling module, which is only heterogeneous in the Basal-like subtype contains the EGFR (epidermal growth factor receptor) gene. EGFR has been implicated in the biology of triple-negative tumors [52]. Unfortunately, it has also been shown that anti-EGFR treatments with the drug cetuximab have limited success against Basal-like tumors [3].

All interpretations based on functional enrichments must be treated with caution as the annotations are far from complete or even up to date. There are also inherent limitations to using modules based on co-expression as genes involved in the same biological process may not share the same expression pattern.

In the immediate future, we would like to refine our measure of modular heterogeneity by further dissection of each module into more biologically meaningful sub-modules. The assessment of the sub-modules’ relative contributions to breast cancer heterogeneity might help identify clinically meaningful sub-subtypes and thus provide more effective personalized medicine. Additionally, we are interested in elucidating the contribution of intra-tumor heterogeneity to modular heterogeneity and if this phenomenon can also be observed at the level of individual clones or single cells. On a more theoretical plane, modular heterogeneity is an appealing framework with which to investigate other tumor properties such as the development of resistance to treatment through robustness and evolvability. We hypothesize that cancer cells can more effectively explore their fitness landscapes by incrementally and combinatorially de-regulating gene modules. Each step taken in the modular progression could provide a rich neutral space in which to tinker. This may allow tumor populations to explore the space of possible trait combinations more efficiently by pruning vast sections of the transcriptome space.

## Conclusion

In this study we have dissected breast cancer heterogeneity and shown (1) that heterogeneity is not only a global property of each tumor subtype transcriptome but that it is concentrated locally in gene modules, (2) that each tumor subtype exhibits a unique combination of modules that are heterogeneous with respect to normal breast tissue, (3) that subtypes of breast cancer can have high local heterogeneity despite having low global heterogeneity and (4) that the number of modules that are heterogeneous when compared to normal tissue increases with subtype aggressiveness. We propose modular heterogeneity as a new view on breast cancer heterogeneity that will help us refine the molecular classification of tumors, assess risk for individual patients and predict response to treatment.

## Electronic supplementary material

Additional file 1: **Table S1.** P-values of 30 pair-wise comparisons of *β*-diversity between all subtypes for the global transcriptome, before and after correction. (ZIP 32 KB)

Additional file 2: **Table S2.** Results of gene ontology analysis for all modules. (ZIP 342 KB)

Additional file 3: **Table S3.** Gene names for all modules. (ZIP 58 KB)

Additional file 4: **Table S4.** P-values of 240 pair-wise comparisons of *β*-diversity between all subtypes for all modules, before (a) and after (b) correction. (ZIP 36 KB)

Additional file 5: **Figure S1.** Box plots corresponding to the patterns of *β*-diversity across subtypes and stages (normal, cancerous and metastatic). The box plots at the top show *β*-diversities for the global transcriptome of each subtype. The box plots at the bottom show *β*-diversities for the eight modules. (ZIP 1 MB)

## References

[1] Cancer Genome, Atlas Network and others (2012). **Comprehensive molecular portraits of human breast tumours**. Nature.

[2] Goldhirsch A, Wood W, Coates A, Gelber R, Thürlimann B, Senn HJ, Panel members (2011). **Strategies for subtypes—dealing with the diversity of breast cancer: highlights of the St Gallen international expert consensus on the primary therapy of early breast cancer 2011**. Ann Oncol.

[3] Higgins MJ, Baselga J (2011). **Targeted therapies for breast cancer**. J Clin Invest.

[4] Polyak K (2011). **Heterogeneity in breast cancer**. J Clin Invest.

[5] Perez EA (2011). **Breast cancer management: opportunities and barriers to an individualized approach**. Oncologist.

[6] Russnes H, Navin N, Hicks J, Borresen-Dale A (2011). **Insight into the heterogeneity of breast cancer through next-generation sequencing**. The J Clin Invest.

[7] Perou CM, Børresen-Dale AL (2011). **Systems biology and genomics of breast cancer**. Cold Spring Harbor Perspect Biol.

[8] Perou CM (2011). **Molecular stratification of triple-negative breast cancers**. Oncologist.

[9] Zelnak AB, O’Regan RM (2013). **Genomic subtypes in choosing adjuvant therapy for breast cancer**. Oncology.

[10] Reis-Filho J, Tutt A (2008). **Triple negative tumours: a critical review**. Histopathology.

[11] Parker JS, Mullins M, Cheang MC, Leung S, Voduc D, Vickery T, Davies S, Fauron C, He X, Hu Z, Quackenbush JF, Stijleman IJ, Palazzo J, Marron JS, Nobel AB, Mardis E, Nielsen TO, Ellis MJ, Perou CM, Bernard PS (2009). **Supervised risk predictor of breast cancer based on intrinsic subtypes**. J Clinical Oncol.

[12] Nielsen TO, Parker JS, Leung S, Voduc D, Ebbert M, Vickery T, Davies SR, Snider J, Stijleman IJ, Reed J, Cheang MC, Mardis ER, Perou CM, Bernard PS, Ellis MJ (2010). **A Comparison of PAM50 intrinsic subtyping with immunohistochemistry and clinical prognostic factors in tamoxifen-treated estrogen receptor–positive breast cancer**. Clin Cancer Res.

[13] Harrell J, Prat A, Parker J, Fan C, He X, Carey L, Anders C, Ewend M, Perou C (2012). **Genomic analysis identifies unique signatures predictive of brain, lung, and liver relapse**. Breast Cancer Res Treat.

[14] Weigelt B, Baehner FL, Reis-Filho JS (2010). **The contribution of gene expression profiling to breast cancer classification, prognostication and prediction: a retrospective of the last decade**. The J Pathol.

[15] Anderson MJ, Ellingsen KE, McArdle BH (2006). **Multivariate dispersion as a measure of beta diversity**. Ecol Lett.

[16] Park SY, Gönen M, Kim HJ, Michor F, Polyak K (2010). **Cellular and genetic diversity in the progression of in situ human breast carcinomas to an invasive phenotype**. J Clin Invest.

[17] Ho JW, Stefani M, dos Remedios CG, Charleston MA (2008). **Differential variability analysis of gene expression and its application to human diseases**. Bioinformatics.

[18] Anderson MJ, Crist TO, Chase JM, Vellend M, Inouye BD, Freestone AL, Sanders NJ, Cornell HV, Comita LS, Davies KF, Harrison SP, Kraft NJ, Stegen JC, Swenson NG (2011). **Navigating the multiple meanings of*****β*****diversity: a roadmap for the practicing ecologist**. Ecol Lett.

[19] Anderson MJ (2006). **Distance-based tests for homogeneity of multivariate dispersions**. Biometrics.

[20] Manly BF (2004). Multivariate statistical methods: a primer.

[21] Manly BF (2006). Randomization, bootstrap and Monte Carlo methods in biology.

[22] Zhang B, Horvath S (2005). **A general framework for weighted gene co-expression network analysis**. Stat Appl Genet Mol Biol.

[23] Horvath S (2011). Weighted Network Analysis: Applications in Genomics and Systems Biology.

[24] Langfelder P, Horvath S (2008). **WGCNA: an R package for weighted correlation network analysis**. BMC Bioinformatics.

[25] Bergmann S, Ihmels J, Barkai N (2003). **Similarities and differences in genome-wide expression data of six organisms**. PLoS Biology.

[26] Ravasz E, Somera A, Mongru D, Oltvai Z, Barabási A (2002). **Hierarchical organization of modularity in metabolic networks**. Science.

[27] Langfelder P, Zhang B, Horvath S (2008). **Defining clusters from a hierarchical cluster tree: the Dynamic Tree Cut package for R**. Bioinformatics.

[28] Voineagu I, Wang X, Johnston P, Lowe JK, Tian Y, Horvath S, Mill J, Cantor RM, Blencowe BJ, Geschwind DH (2011). **Transcriptomic analysis of autistic brain reveals convergent molecular pathology**. Nature.

[29] Farber CR, Bennett BJ, Orozco L, Zou W, Lira A, Kostem E, Kang HM, Furlotte N, Berberyan A, Ghazalpour A, Suwanwela J, Drake TA, Eskin E, Wang QT, Teitelbaum SL, Lusis AJ (2011). **Mouse genome-wide association and systems genetics identify Asxl2 as a regulator of bone mineral density and osteoclastogenesis**. PLoS Genetics.

[30] Eden E, Navon R, Steinfeld I, Lipson D, Yakhini Z (2009). **GOrilla: a tool for discovery and visualization of enriched GO terms in ranked gene lists**. BMC Bioinformatics.

[31] Eisen M, Spellman P, Brown P, Botstein D (1998). **Cluster analysis and display of genome-wide expression patterns**. Proc Nat Acad Sci.

[32] Oldham M, Horvath S, Geschwind D (2006). **Conservation and evolution of gene coexpression networks in human and chimpanzee brains**. Proc Nat Acad Sci.

[33] Miller J, Horvath S, Geschwind D (2010). **Divergence of human and mouse brain transcriptome highlights Alzheimer disease pathways**. Proc Nat Acad Sci.

[34] Hawrylycz MJ, Lein S, Guillozet-Bongaarts AL, Shen EH, Ng L, Miller JA, van de Lagemaat LN, Smith KA, Ebbert A, Riley ZL, Abajian C, Beckmann CF, Bernard A, Bertagnolli D, Boe AF, Cartagena PM, Chakravarty MM, Chapin M, Chong J, Dalley RA, Daly BD, Dang C, Datta S, Dee N, Dolbeare TA, Faber V, Feng D, Fowler DR, Goldy J, Gregor BW (2012). **An anatomically comprehensive atlas of the adult human brain transcriptome**. Nature.

[35] Horvath S, Zhang B, Carlson M, Lu K, Zhu S, Felciano R, Laurance M, Zhao W, Qi S, Chen Z, Lee Y, Scheck AC, Liau LM, Wu H, Geschwind DH, Febbo PG, Kornblum HI, Cloughesy TF, Nelson SF, Mischel PS (2006). **Analysis of oncogenic signaling networks in glioblastoma identifies ASPM as a molecular target**. Proc Nat Acad Sci.

[36] Gargalovic PS, Imura M, Zhang B, Gharavi NM, Clark MJ, Pagnon J, Yang WP, He A, Truong A, Patel S, Nelson SF, Horvath S, Berliner JA, Kirchgessner TG, Lusis AJ (2006). **Identification of inflammatory gene modules based on variations of human endothelial cell responses to oxidized lipids**. Proc Nat Acad Sci.

[37] Iancu O, Darakjian P, Walter N, Malmanger B, Oberbeck D, Belknap J, McWeeney S, Hitzemann R (2010). **Genetic diversity and striatal gene networks: focus on the heterogeneous stock-collaborative cross (HS-CC) mouse**. BMC Genomics.

[38] Feinberg A, Ohlsson R, Henikoff S (2006). **The epigenetic progenitor origin of human cancer**. Nat Rev Genet.

[39] Lupien M, Eeckhoute J, Meyer CA, Wang Q, Zhang Y, Li W, Carroll JS, Liu XS, Brown M (2008). **FoxA1 translates epigenetic signatures into enhancer-driven lineage-specific transcription**. Cell.

[40] Sharma S, Kelly T, Jones P (2010). **Epigenetics in cancer**. Carcinogenesis.

[41] Akhtar-Zaidi B, Cowper-Sal ·lari R, Corradin O, Saiakhova A, Bartels CF, Balasubramanian D, Myeroff L, Lutterbaugh J, Jarrar A, Kalady MF, Willis J, Moore JH, Tesar PJ, Laframboise T, Markowitz S, Lupien M, Scacheri PC (2012). **Epigenomic enhancer profiling defines a signature of colon cancer**. Science.

[42] Johnston SR (2010). **New strategies in estrogen receptor–positive breast cancer**. Clinical Cancer Res.

[43] Brisken C, Heineman A, Chavarria T, Elenbaas B, Tan J, Dey SK, McMahon JA, McMahon AP, Weinberg RA (2000). **Essential function of Wnt-4 in mammary gland development downstream of progesterone signaling**. Genes & Dev.

[44] Gestl SA, Leonard TL, Biddle JL, Debies MT, Gunther EJ (2007). **Dormant Wnt-initiated mammary cancer can participate in reconstituting functional mammary glands**. Mol Cell Biol.

[45] Garay JP, Park BH (2012). **Androgen receptor as a targeted therapy for breast cancer**. Am J Cancer Res.

[46] Cowper-Sal ·lari R, Zhang X, Wright JB, Bailey SD, Cole MD, Eeckhoute J, Moore JH, Lupien M (2012). **Breast cancer risk-associated SNPs modulate the affinity of chromatin for FOXA1 and alter gene expression**. Nat Genet.

[47] Santana-Davila R, Perez EA (2010). **Treatment options for patients with triple-negative breast cancer**. J Hematol Oncol.

[48] Bianchini G, Qi Y, Alvarez RH, Iwamoto T, Coutant C, Ibrahim NK, Valero V, Cristofanilli M, Green MC, Radvanyi L, Hatzis C, Hortobagyi GN, Andre F, Gianni L, Symmans WF, Pusztai L (2010). **Molecular anatomy of breast cancer stroma and its prognostic value in estrogen receptor–positive and–negative cancers**. J Clinical Oncol.

[49] Desmedt C, Haibe-Kains B, Wirapati P, Buyse M, Larsimont D, Bontempi G, Delorenzi M, Piccart M, Sotiriou C (2008). **Biological processes associated with breast cancer clinical outcome depend on the molecular subtypes**. Clinical Cancer Res.

[50] Rody A, Holtrich U, Pusztai L, Liedtke C, Gaetje R, Ruckhaeberle E, Solbach C, Hanker L, Ahr A, Metzler D, Engels K, Karn T, Kaufmann M (2009). **T-cell metagene predicts a favorable prognosis in estrogen receptor-negative and HER2-positive breast cancers**. Breast Cancer Res.

[51] Rugo HS (2012). **Inhibiting angiogenesis in breast cancer: the beginning of the end or the end of the beginning?**. J Clinical Oncol.

[52] Anders C, Carey LA (2008). **Understanding and treating triple-negative breast cancer**. Oncology (Williston Park, NY).

