# Supplementary material for: Combining functional genomics strategies identifies modular heterogeneity of breast cancer intrinsic subtypes
Source: BioData Min. 2014 Dec 29;7:27. doi: 10.1186/1756-0381-7-27 (PMC4350320; doi:10.1186/1756-0381-7-27)
Supplement: Supplementary file 5 [file 13040_2013_126_MOESM5_ESM.zip › xl/media/image3.pdf]

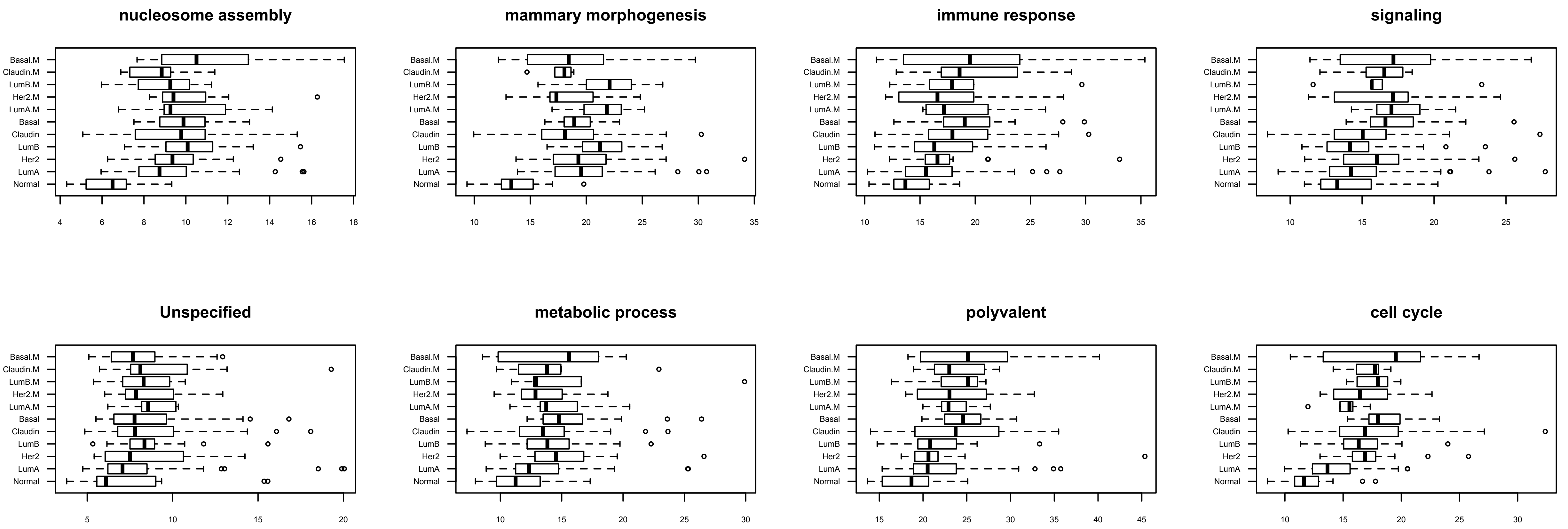

nucleosome assembly
mammary morphogenesis
immune response
signaling
Unspecified
metabolic process
polyvalent
cell cycle
Basal.M
Claudin.M
LumB.M
Her2.M
LumA.M
Basal
Claudin
LumB
Her2
LumA
Normal
